# Overview and development trend of non-pharmacological therapies for poststroke gait abnormalities in the past decade: A bibliometric study

**DOI:** 10.1097/MD.0000000000045763

**Published:** 2025-10-31

**Authors:** Xianggang Meng, Junfeng Zhang, Hao Chen, Yan Guo, Mengying Rong, Yuetong Li, Yuzheng Du, Guiping Li, Chen Li

**Affiliations:** a First Teaching Hospital of Tianjin University of Traditional Chinese Medicine, Tianjin, China; b National Clinical Research Center for Chinese Medicine Acupuncture and Moxibustion, Tianjin, China; c Tianjin Academy of Traditional Chinese Medicine Affiliated Hospital, Tianjin, China; d Tianjin Medical University, Tianjin, China; e Tianjin University of Traditional Chinese Medicine, Tianjin, China; f Tianjin University of Traditional Chinese Medicine Second Affiliated Hospital, Tianjin, China.

**Keywords:** bibliometric, gait, non-pharmacological therapy, rehabilitation, stroke, visualization analysis

## Abstract

**Background:**

Stroke survivors typically show decreased walking ability, and restoring walking ability is the main goal of poststroke rehabilitation. Non-pharmacological therapies provide stroke patients with diverse and personalized rehabilitation options. However, the development process and research trends in this field have not yet been explored and organized.

**Objective:**

Summarize the research hotspots and development trends of non-pharmacological therapies for poststroke gait abnormality in the past 10 years, and provide a basis and feasible suggestions for formulating systematic diagnosis and treatment plans for poststroke gait abnormality.

**Method:**

Retrieve literature related to stroke and gait from the Web of Science in the past 10 years, and use CiteSpace and VOSviewer to conduct statistical analysis on the number of articles publications, research strength, and keywords.

**Results:**

A total of 4468 articles were retrieved, and 965 articles that met the criteria were included after screening. The results showed that the number of publications has generally increased in the past 10 years, with research teams and institutions in East Asia, North America, and Europe are the main research forces in the field. A total of 2482 keywords were involved, forming 14 larger clusters. Current research mainly focuses on non-pharmacological therapies to improve gait ability and motor function, especially in improving balance, velocity, and motor performance.

**Conclusion:**

This study found the following research hotspots: the development of technology-assisted rehabilitation; the effectiveness of multimodal rehabilitation interventions; and research on the mechanism of neuroplasticity. Non-pharmacological therapies have significant potential in improving gait abnormalities. Conducting standardized randomized controlled trials to improve the reliability and validity of research, combined with individualized treatment plans and emerging technologies, is expected to further enhance the overall level of gait rehabilitation and motor function in the future.

## 1. Introduction

Stroke is characterized by a high incidence rate, high recurrence rate, high mortality rate, high disability rate, and significant economic burden.^[1]^ According to the 2019 Global Burden of Diseases study, stroke is the 2nd leading cause of death and the 3rd leading cause of disability globally.^[2]^ Approximately 50% to 60% of stroke patients exhibit persistent gait abnormalities.^[3]^ The primary characteristics of gait abnormalities after stroke are gait instability and asymmetry.^[4]^ Patients often experience shortened stride length, slowed velocity, and reduced cadence, which may be accompanied by abnormal postures such as dragging, external rotation, or inversion of the affected lower limb.^[5]^ Gait abnormalities not only increase the risk of patients falling, but may also lead to secondary problems such as joint wear and pain.^[6–8]^ Additionally, gait abnormalities may also affect the patient’s psychological state, causing negative emotions such as depression and anxiety, further affecting their rehabilitation process.^[9,10]^ Gait abnormalities not only have a serious impact on the quality of life and workability, but also often require a large amount of medical resources and social support during the rehabilitation process, imposing a heavy burden on the family and social medical system.^[11,12]^

To effectively improve poststroke gait abnormalities, non-pharmacological therapies based on physical therapy have been used in rehabilitation for a long time.^[13,14]^ Non-pharmacological therapies, including electrical stimulation, mirror therapy, and constraint-induced movement therapy have been recognized.^[15–17]^ In recent years, with the advancement of medical technology and the renewal of rehabilitation concepts, a variety of novel non-pharmacological treatment methods have emerged,^[18–21]^ broadening the range of non-pharmacological therapies, including exoskeleton, virtual reality (VR), transcranial magnetic stimulation (TMS), robotic-assisted therapy (RAT), and transcranial direct current stimulation (tDCS). Non-pharmacological therapy has the advantages of noninvasive and low-risk. It effectively enhances patients’ walking abilities, restores gait coordination and symmetry,^[22–26]^ provides more diverse and personalized rehabilitation options for survivors, and demonstrates the unique advantages and application prospects of non-pharmacological therapy. However, the development process and research trends in this field have not yet been explored and organized.

Bibliometric analysis can integrate the strengths of multiple disciplines and transform initially isolated technologies and methodologies into a holistic framework,^[27]^ which makes it easier for researchers to understand the development process and research frontiers of this field. Therefore, researchers can gain insights into the challenges and opportunities inherent in future research. CiteSpace and VOSviewer, with distinct algorithms and focal points, are commonly utilized software in bibliometrics analysis, complementing each other effectively in the creation of Knowledge maps.^[28–30]^ Using bibliometric methods, systematically combed and deeply analyzing the research results of non-pharmacological therapies in promoting gait rehabilitation poststroke is of significance for guiding clinical practice and advancing disciplinary development.

This study analyzed the literature on non-pharmacological therapies for poststroke gait abnormalities through bibliometrics and visualization analysis, to provide a scientific basis and reference for non-pharmacological treatment options, and promote the widespread application and in-depth development of non-pharmacological therapies in the field of stroke gait rehabilitation. We also hope to reveal the research trends and frontier directions in this field from the perspective of bibliometrics, and provide new insights and directions for future research.

## 2. Data and methods

### 2.1. Data collection

**Figure 1.**
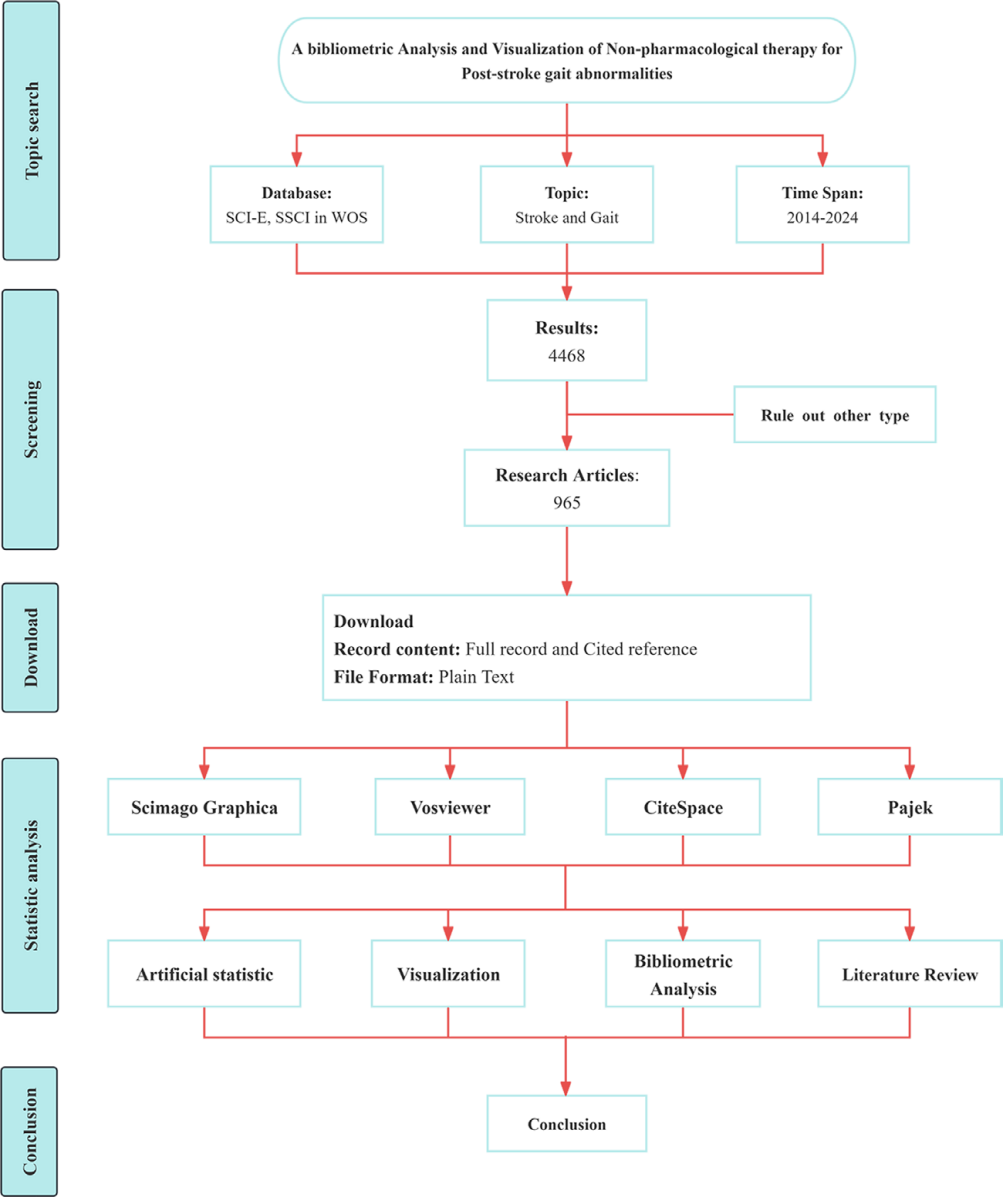
Flow chart of bibliometric analysis. SCI-E = Science Citation Index Expanded, SSCI = Social Sciences Citation Index, WOS = Web of Science.

Web of Science (WOS) is the data source for this study, with indexes selected Science Citation Index Expanded and Social Sciences Citation Index. To ensure the comprehensive and accuracy of the retrieved data, TS = (“stroke” AND “gait”) was used as the search formula, the language was English, the literature type was Article, the time was 2014 to 2024, and the retrieval deadline was June 12, 2024. A total of 4468 articles were obtained. Two independent researchers screened the studies on non-pharmacological therapies to improve poststroke gait in the obtained literature, and the 2 discussed and resolved the inconsistencies. If the inconsistency still exists, the final decision will be made by an additional author. After screening and cleaning each literature, a total of 965 valid articles were obtained. The bibliometric research process is shown in Figure 1.

### 2.2. Data analysis

CiteSpace 6.1.R6 software was used to conduct co-occurrence analysis on authors, institutions, keywords, and literature sources, and draw relevant Knowledge maps. The software time parameter was set to span from January 2014 to December 2024, the time slice was set to 1 year. The node types were selected as authors, institutions, and keywords. The pruning type selected was Pathfinder, Pruning sliced networks, and Pruning merged networks as pruning types. VOSviewer 1.6.2 software was used for co-citation analysis. National publication analysis was completed using VOSviewer combined with Scimago Graphica software.

## 3. Result

### 3.1. Literature screening results

After screening through the above method, 965 articles were included from 3930 authors from 1491 institutions in 58 countries, published in 170 journals, and cited 17,271 articles from 3534 journals.

### 3.2. Analysis of journal publication trends

**Figure 2.**
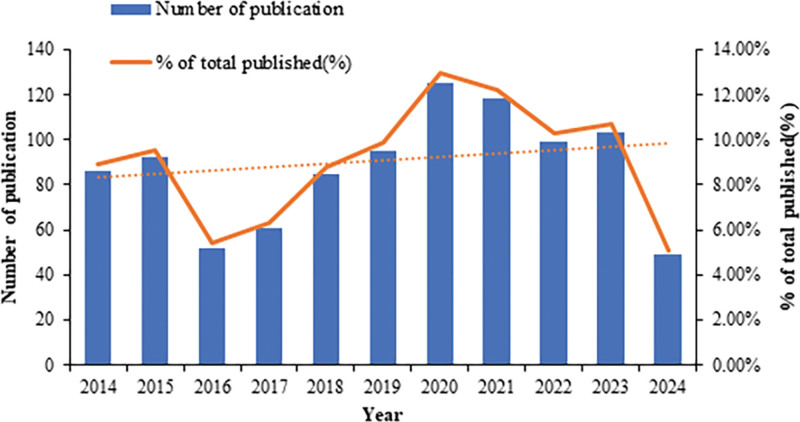
Annual publication volume and publication ratio.

Figure 2 shows the time distribution of papers published in the field of non-pharmacological therapies for poststroke gait abnormalities. The growth of publications on this topic is divided into 2 stages based on 2016. The year 2016 was with the lowest number of articles published in the past decade. In 2016, the Journal of Physical Therapy Science, sponsored by the Japan Science and Technology Information Aggregator, was suppressed by Thomson Reuters and was not included in the Science Citation Index journal of rehabilitation science.^[31]^ This led to changes in the composition of core journals in this field and a low point of publication. After 2016, the number of articles published continued to rise. After 2019, the number of articles published has remained stable at more than 95 and reached a peak in 2020 (125 articles). Subsequently, perhaps due to the impact of the COVID-19 pandemic, scholars’ attention to in-hospital rehabilitation of stroke declined to a certain extent, but after the main impact of the epidemic ended, the number of publications continued to rise.

### 3.3. National collaboration network analysis

**Figure 3.**
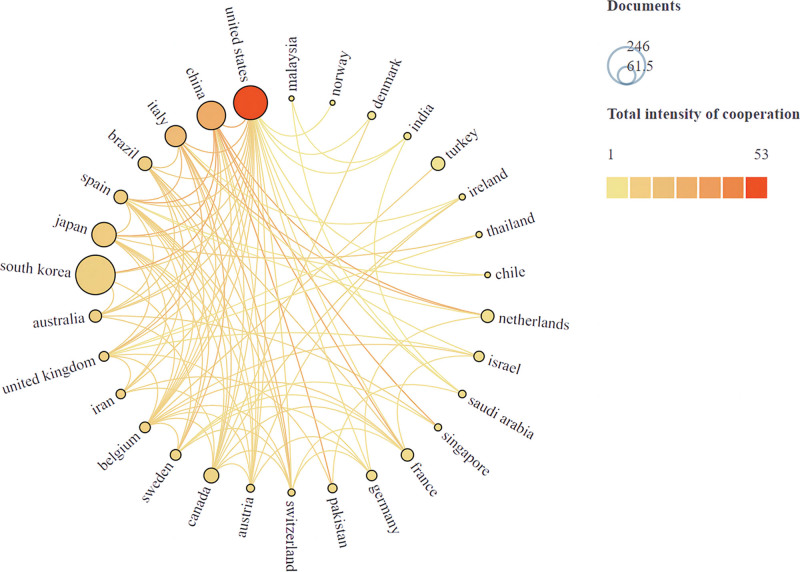
Knowledge map of the number of publications from countries and the intensity of cooperation between countries. The larger the circular node, the more publications it has, and the redder the node color, the more collaborative research publications it has with other countries.

This study analyzed the publication volume of 58 countries. VOSviewer combined with Scimago Graphica software was used to conduct a visualization analysis of countries with more than 6 publications in this field and the intensity of cooperation between countries. The results are shown in Figure 3. The distribution of publications in this field is uneven among countries, and the top effect is very significant. Most papers were published by scholars from a few countries. To further analyze the countries with high publication rates in this field, Table 1 presents the top 10 countries with the most publications in this field. From the table data analysis, it can be seen that Korean scholars have contributed the most research papers in this field (246 in total), but the average citation rate of the documents is low, at 12.7. Followed by the United States, with a total of 181 publications and 3156 citations. Spain had the highest number of citations per paper, with 29 papers receiving 1008 citations, and an average of 34.8 citations per paper. The close cooperation among countries in this field highlights that improving hemiplegic gait is a global challenge in this field. East Asian countries such as South Korea, China, and Japan have published relatively more papers, but the number of citations is insufficient, indicating that the quality of research literature in these countries is relatively low. Developed countries account for a large proportion of the total number of papers, which may be related to the large amount of funds invested in scientific research in developed countries and the high incidence of stroke in developed countries.^[2]^

**Table 1:** Top 10 countries by publication volume.

Rank	Country	Publications	Citations	Average citation/publication
1	South Korea	246	3123	12.7
2	United States	181	3156	17.4
3	China	128	1229	9.6
4	Japan	94	1128	12.0
5	Italy	70	1412	20.2
6	Canada	35	478	13.7
7	Brazil	30	302	10.1
8	Spain	29	1008	34.8
9	Turkey	29	271	9.3
10	Netherlands	26	360	13.8

### 3.4. Institutional collaboration network analysis

**Figure 4.**
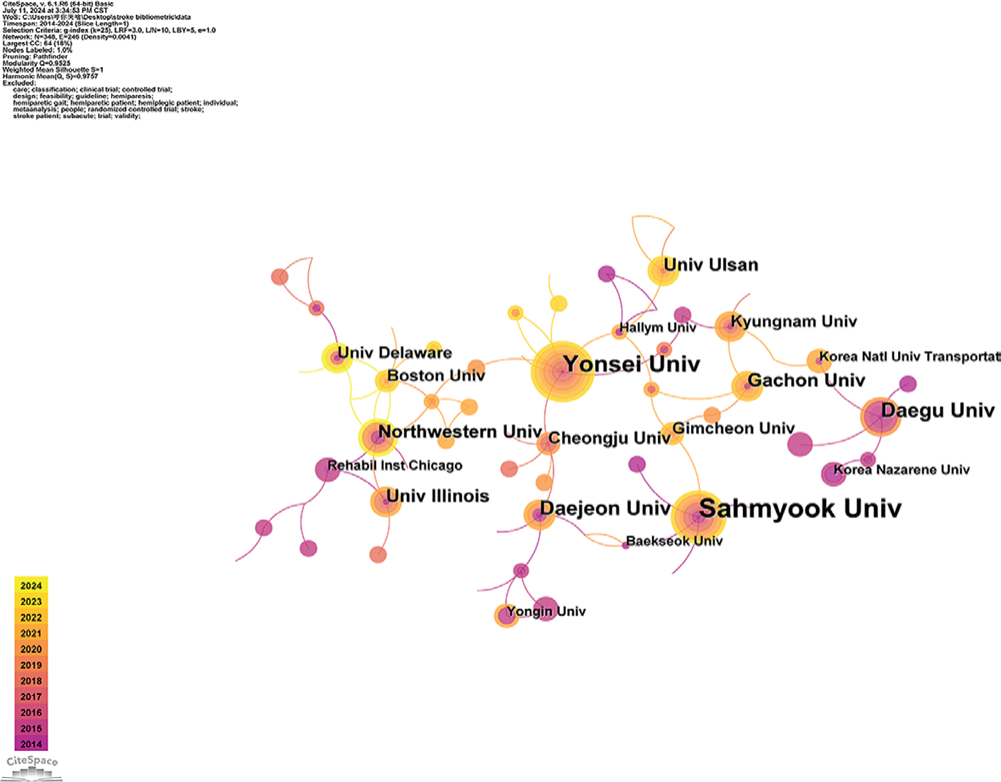
Research institution collaboration knowledge map.

VOSviewer was used to draw a research institution cooperation map based on literature data, as shown in Figure 4. Table 2 lists the top 10 high-publishing institutions. Among the high-publishing institutions, there are 7 South Korean institutions, 2 American institutions, and 1 Japanese institution. Northwestern University in the United States is the institution with the highest average citation. In the collaborative network of research institutions, Sahmyook University, Yonsei University, Daegu University, Daejeon University, Gachon University, Kyungnam University, and University Ulsan have good institutional cooperation. Research institutions centered around Yonsei University and Gachon University have collaborated extensively in the field of robotics. Research institutions led by Sahmyook University and Daejeon University have conducted in-depth studies in the field of walking ability. From Table 2, it can be seen that South Korean institutions have a leading position in the number of publications in this field, but the United States institutions have a greater influence in this field.

**Table 2:** Top 10 Institutions by publication volume.

Rank	Institution	Country	Publications	Citations	Average citation/publication
1	Sahmyook University	South Korea	49	869	17.7
2	Yonsei University	South Korea	31	233	7.5
3	Daegu University	South Korea	19	183	9.6
4	Daejeon University	South Korea	19	204	10.7
5	Northwestern University	United States	18	520	28.9
6	Gachon University	South Korea	17	155	9.1
7	University Illinois	United States	17	240	14.1
8	Fujita Hlth University	Japan	16	141	8.8
9	Kyungnam University	South Korea	14	116	8.3
10	University Ulsan	South Korea	14	179	12.8

### 3.5. Author analysis

Table 3 shows the top 10 highly productive authors in this field. Among the highly productive authors, Yang Yea-Ru has the most publications. From 2014 to June 2024, he published a total of 11 papers, received 188 citations, and the average number of citations per paper was 17.1. The 2nd is Wang Ray-Yau, with 10 papers and 177 citations, and the average number of citations per article was 17.7. Both scholars work at the Department of Physical Therapy and Assistive Technology, National Yang-Ming University, Taiwan, China. Based on the analysis of published literatures, the 2 scholars are more concerned with the improvement of gait under walking and cognitive dual tasks by non-pharmacological therapies,^[32,33]^ and have applied different types of gait training extensively.^[34–36]^

**Table 3:** Top 10 authors by number of publications.

Rank	Author	Documents	Citations	Average citation/publication
1	Yang Yea-Ru	11	188	17.1
2	Wang Ray-Yau	10	177	17.7
3	Chung Yijung	10	160	16
4	Chun Min Ho	10	64	6.4
5	Calabro Rocco Salvatore	9	233	25. 9
6	Awad Louis N	9	161	17. 9
7	Saitoh Eiichi	9	100	11.1
8	Cha Yong-Jun	9	65	7.2
9	Cynn Heon-seock	9	50	5. 6
10	Franceschini Marco	8	129	16.1

**Figure 5.**
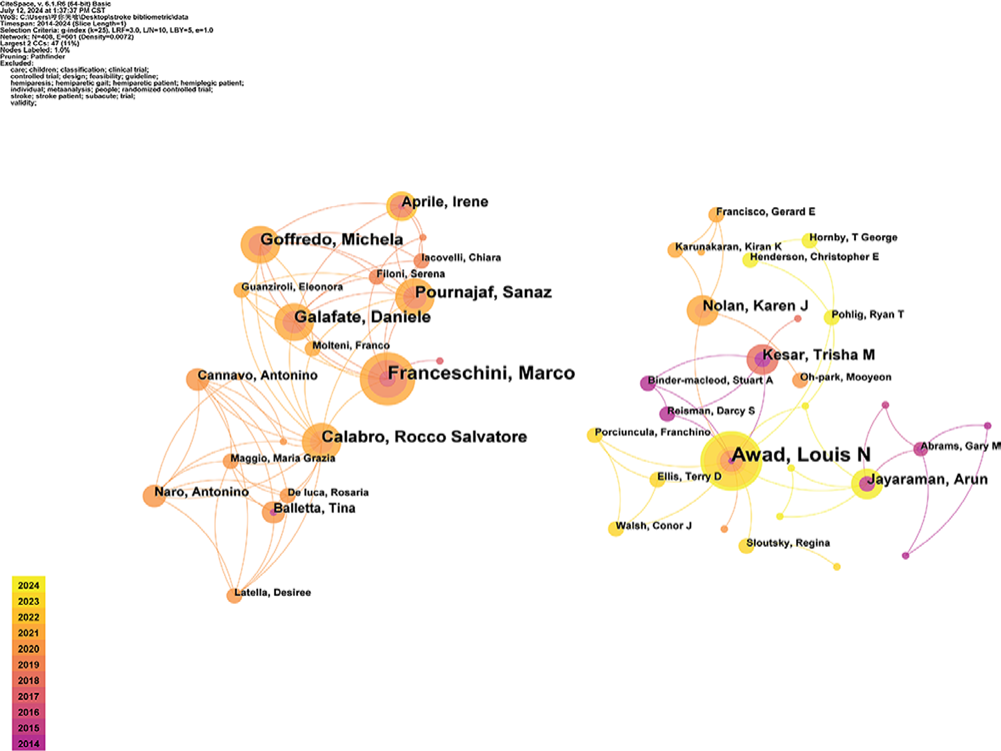
Cooperation map of authors.

Further analysis of the author’s collaboration with the main research teams. As shown in Figure 5, scholars in this field mainly establish collaborative relationships with highly productive authors as the core. The most prominent sub-network is composed of the Franceschini Marco and Calabro Rocco Salvatore teams with high publication volume and high relevance. The team has collaborated extensively in the field of RAT in the past 5 years. Another prominent author collaboration network is composed of Awad Louis N as the core which has collaborated extensively over the past 10 years, mainly in the field of gait biomechanics. Except for the above authors, most of the other authors conduct related research in the form of smaller teams or independent individuals, resulting in insufficient collaboration among authors.

### 3.6. Literature source analysis

Statistics on the journals to which the literature belongs show that in the past decade, except for a small number of comprehensive journals, most of the journals published in this field belong to neurorehabilitation. Table 4 shows the top 10 journals with the highest number of articles published. Among them, Journal of Neuroengineering and Rehabilitation, Brain Sciences, and Frontiers in Neurology are open-access journals, indicating that the strong development of open-access journals in recent years has strongly promoted the research progress in this field. Although the implementation method of open access is still debated by scholars, the idea of free sharing of research results has been widely recognized.^[37]^ The journal with the highest average number of citations per article is the Journal of Neuroengineering and Rehabilitation in the field of neuroscience and biomedical engineering, with a total of 1597 articles published and 24.57 citations per article. This shows that the literature published in this journal is high quality and has received widespread attention in the field of poststroke gait rehabilitation. The main research design of the literature published in this journal is randomized controlled trials (RCTs), focusing on the use of RAT, bioelectric stimulation, and VR. This indicates that in the field of poststroke gait rehabilitation, the above technologies are still the focus of core journals.

**Table 4:** Top 10 journals by publication volume.

Rank	Source	Publications	Citations	Average citation/publication
1	Journal of Physical Therapy Science	66	1044	15.82
2	Journal of Neuroengineering and Rehabilitation	65	1597	24.57
3	Neurorehabilitation	56	718	12.82
4	Topics in Stroke Rehabilitation	38	469	12.34
5	International Journal of Rehabilitation Research	34	362	10.65
6	Brain Sciences	31	220	7.10
7	Clinical Rehabilitation	30	743	24.77
8	Journal of Stroke & Cerebrovascular Diseases	29	443	15.28
9	Frontiers in Neurology	28	127	4.54
10	Gait & Posture	26	325	12.50

### 3.7. Keyword analysis

#### 3.7.1. Co-occurrence analysis

**Figure 6.**
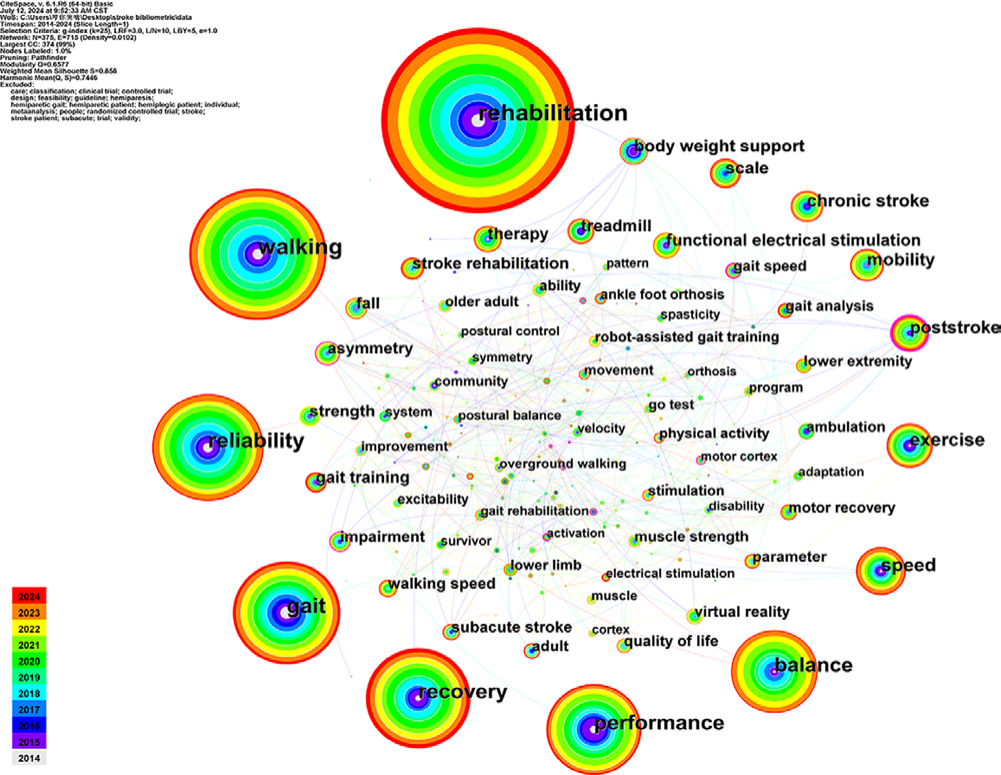
Keywords co-occurrence network. The larger the circular nodes in the figure, the more frequently the keywords appear, and the more they represent the hot spots in the field. The node connections represent the strength of the association. The thicker the lines, the more times the 2 appear in the same literature.

Keywords are the condensed version of the core content of the article. High-frequency keywords are usually used to show the hot research content in the field. We used CiteSpace to visualize the co-occurrence of 2482 keywords included in the literature, as shown in Figure 6. The top 10 high-frequency keywords and their connection strengths are extracted, as shown in Table 5. As can be seen from Figure 6 and Table 5, high-frequency keywords such as rehabilitation, walking, and reliability constitute the representative terms in this field.

**Table 5:** Top10 keywords in the field of non-pharmacological therapies for poststroke gait rehabilitation.

Rank	Count	Centrality	Keywords
1	343	0.01	Rehabilitation
2	247	0.02	Walking
3	202	0.01	Reliability
4	195	0.01	Gait
5	190	0.02	Recovery
6	172	0.01	Performance
7	158	0.02	Balance
8	95	0.01	Speed
9	87	0.02	Exercise
10	70	0.07	Poststroke

#### 3.7.2. Cluster analysis

**Figure 7.**
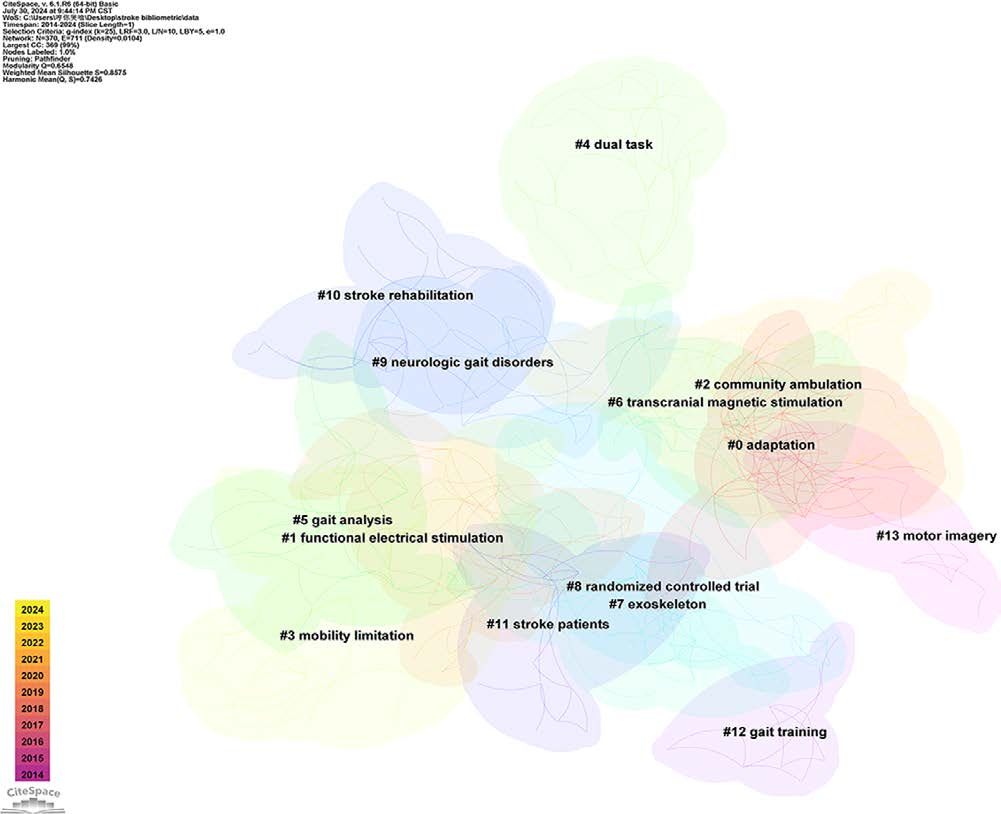
Cluster view of keywords clustering analysis. Different color clusters represent different keyword groups.

Use Citespace’s “K” clustering-LLR algorithm to perform cluster analysis on keywords. The result is shown in Figure 7. The analysis shows that a total of 14 large clusters are formed, and the overlap and interaction between clusters indicate that the connections between clusters are closer. Modularity *Q* = 0.6548 > 0.3, indicating a clear and significant clustering structure, and mean Silhouette *S* = 0.8575 > 0.7, indicating reasonable clustering and high credibility.^[38]^

Clusters 1, 2, 4, 6, 7, 12, and 13 reflect the main non-pharmacological therapies currently used to promote poststroke gait rehabilitation, including functional electrical stimulation (FES), community ambulation, dual task, TMS, exoskeleton, gait training, motor imagery. These methods have accumulated theoretical and practical foundations for many years, and a large number of researchers are engaged in related work. In the future, the relevant topics in these clusters will continue to dominate research and may give rise to new clusters in the field. Cluster 0 represents the possible mechanism of non-pharmacological therapies promoting gait rehabilitation, which is related to adaptation change. Clusters 3 and 9 focus on the overall performance of gait abnormalities, including mobility limitation and neurologic gait disorders. The literature of these 2 clusters also focuses on rehabilitation methods such as resistance training and RAT.

Cluster 5 mainly emphasizes the assessment of gait abnormalities. In addition to traditional gait analysis, new evaluation methods such as motion analysis systems have also been applied in clinical and scientific research.^[39]^ Although clinicians currently use more convenient and fast clinical qualitative observation methods,^[40]^ limited by the subjectivity of observation methods and the inability to quantitatively calculate spatiotemporal parameters, methods such as motion analysis systems will be the mainstream methods for future clinical gait analysis.^[41]^ The formation of cluster 8 indicates that the current research methods in this field are still mainly based on RCTs. As high-level evidence-based medicine, RCTs provide a large amount of clinical evidence in this field and will continue to be the main research paradigm in the future. Clusters 10 and 11 focus on a broad population of stroke patients and stroke rehabilitation.

### 3.8. Co-citation analysis of literature

**Figure 8.**
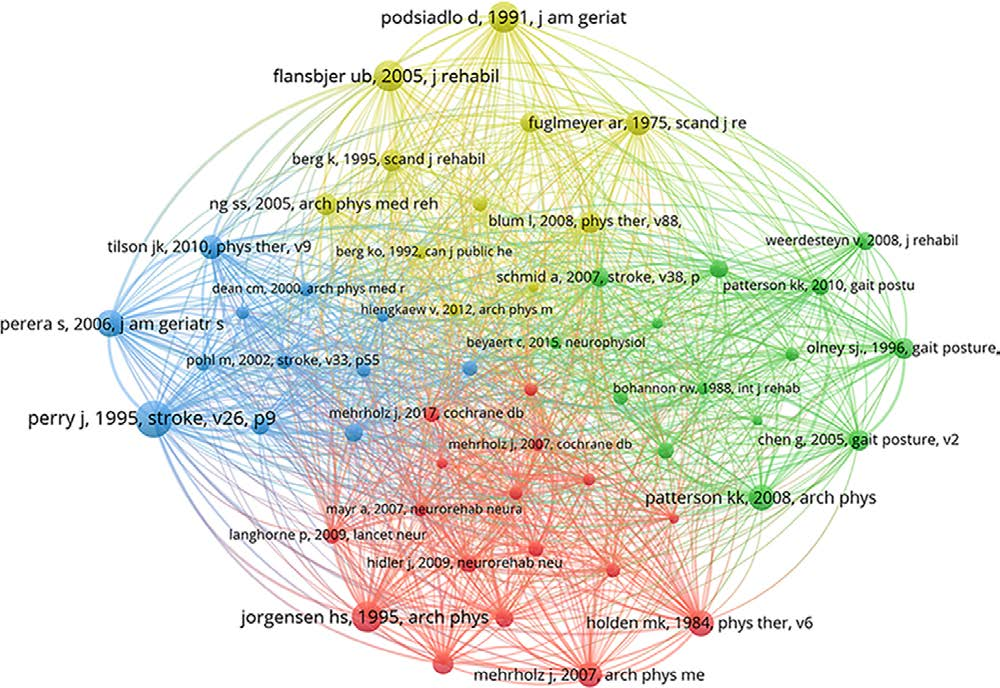
The co-citation relationship map presented by VOSviewer combined with Pajek. Nodes represent literatures, lines indicate co-citation relationships, and different colors represent different literature clusters.

Further analysis of the co-citation of the literature revealed the top 10 most cited literature in this field from 2014 to 2024, as shown in Table 6. These literatures are all fundamental literature for the analysis, measurement, and staging of gait abnormalities after stroke and are of great significance. The literature with the highest number of co-citations was published by Perry J in 1995.^[42]^ In this study, the author provided a quantitative assessment tool for walking ability, which improved the accuracy of predicting patients’ walking ability. Therefore, it occupies an important position in the co-citation literature. Further analysis of the years of the highly cited literature revealed that the majority of them were published between 1990 and 2010, and only 10 articles published after 2010 were cited more than 30 times. This indicates that studies published between 1990 and 2010 constitute the main knowledge foundation in this field. The co-citation relationship map presented by VOSviewer combined with Pajek software is shown in Figure 8. These highly cited articles are divided into 4 clusters. The blue and yellow clusters focus on gait and physical function measurement, as well as the efficacy of early physical therapy. The red and green clusters are mostly review and systematic review studies. The citations of these papers mainly provide support for research on gait analysis, standards, and guidelines for stroke rehabilitation.

**Table 6:** Top10 Most important co-cited publications.

Rank	Title	Source	Year	Co-citation
1	Classification of walking handicap in the stroke population	Stroke	1995	105
2	Recovery of walking function in stroke patients: the Copenhagen Stroke Study	Archives of Physical Medicine And Rehabilitation	1995	90
3	Reliability of gait performance tests in men and women with hemiparesis after stroke	Journal of Rehabilitation Medicine	2005	88
4	The timed “Up & Go”: a test of basic functional mobility for frail elderly persons	Journal of The American Geriatrics Society	1991	88
5	Meaningful change and responsiveness in common physical performance measures in older adults	Journal of The American Geriatrics Society	2006	78
6	Gait asymmetry in community-ambulating stroke survivors	Archives of Physical Medicine And Rehabilitation	2008	76
7	Clinical gait assessment in the neurologically impaired. Reliability and meaningfulness	Physical Therapy & Rehabilitation Journal	1984	75
8	The poststroke hemiplegic patient. 1. A method for evaluation of physical performance	Scandinavian Journal of Rehabilitation Medicine	1975	73
9	Predictive validity and responsiveness of the functional ambulation category in hemiparetic patients after stroke	Archives of Physical Medicine And Rehabilitation	2007	71
10	Meaningful velocity improvement during the first 60 d poststroke: minimal clinically important difference	Physical Therapy & Rehabilitation Journal	2010	68

## 4. Discussion

### 4.1. Research overview

WOS is a high-quality digital literature resource database that covers a large number of literature in different fields. It has been accepted by many researchers and is considered the most suitable database for bibliometric analysis. Therefore, this study selected WOS as the source of literature data.^[43,44]^ The results of this study show that the number of research in this field is steadily increasing, with research teams and institutions in East Asia, North America, and Europe being the main research forces in the field. Current research focuses on non-pharmacological therapies to improve gait rehabilitation and motor function, especially in improving balance, velocity, and motor performance. At the same time, we found that RCTs have played a key role in research in this field, particularly in evaluating the reliability and effectiveness of non-pharmacological therapies.

### 4.2. Analysis of research strength

The research strength of non-pharmacological therapies for poststroke gait abnormalities is increasing. The results of this study show that the research strength is mainly concentrated in several leading research institutions and academic groups including Sahmyook University, Yonsei University, and Daegu University in South Korea, Northwestern University, and University Illinois in the United States, Kyoto University and Fujita Hlth University in Japan. These institutions have published a large number of high-impact papers in this field, and their research results have had an important impact on the international academic community, playing a key role in promoting the research and application of non-pharmacological therapy.

In this study, we found significant regional differences in research in this field. Research activities were relatively intensive in North America, East Asia, and Europe, while research in other regions was relatively sparse. This may be due to the advantages of North America, East Asia, and Europe in terms of resources and funding, medical systems and policies, academic environment, health burden, and disease priority, as well as socioeconomic factors.^[45,46]^ For example, the National Institutes of Health in the United States provides significant research funding, promoting multidisciplinary collaboration including neuroscience, sports medicine, rehabilitation engineering, and psychology.^[47,48]^ National Institutes of Health provides researchers with abundant resources and research platforms, promotes the accumulation of evidence-based evidence, provides evidence support for clinical practice, and encourages researchers to explore new non-pharmacological intervention strategies and rehabilitation methods.^[49,50]^ Similarly, the Japan Society for the Promotion of Science,^[51,52]^ the National Key Research and Development Program of China,^[53]^ and the National Natural Science Foundation of China^[54]^ have all provided support for research in the field of non-pharmacological therapies for poststroke gait abnormalities. Relatively speaking, there is less research activity in other regions because they may face more challenges in the aforementioned aspects,^[55,56]^ such as South America, Central Asia, and Southeast Asia. In the future, increasing medical assistance to these regions, opening up international research funding applications projects, improving medical resources and policy support, promoting international cooperation and other measures will help improve the level of research and rehabilitation worldwide.^[57]^

Some researchers stand out due to their high-yield research results and contributions to the field. For example, scholars from Taiwan, China Yang Yea-Ru, Wang Ray-Yau, and South Korean scholars Chung Yijung and Chun Min Ho are the most productive scholars in this field. Yang Yea-Ru and Wang Ray-Yau achieved good therapeutic effects in the rehabilitation of poststroke gait abnormalities patients using gait training combined with task-oriented electromyography biofeedback or other electrical stimulation devices.^[33,58]^ In addition to focusing on the efficacy of FES,^[59]^ Chung Yijung also found that fast-tempo rhythmic auditory stimulation combined with visual feedback can significantly improve patients’ walking ability.^[60,61]^ Chun Min Ho focused on RAT, and combined with neuroimaging technology, found that the mechanism of RAT may be related to increased cortical activity in stroke patients.^[62–64]^

Some scholars have gradually formed stable research teams due to academic needs, resource sharing, and interdisciplinary cooperation. This study found that 2 stable research teams have been formed in this field. The core authors of the team are from the United States and Italy. American scholars Awad Louis N and Kesar Trisha M, as the core of the team, have published multiple research results in the fields of RAT and real-time gait biofeedback.^[65–70]^ In addition, a stable author collaboration team has been formed with Italian scholars Franceschini Marco and Calabro Rocco Salvatore as the core. These researchers have conducted extensive research on technologies such as RAT and powered exoskeletons and found that these technologies can significantly improve motion tasks compared to conventional rehabilitation training, and have a certain effect on improving spatiotemporal gait parameters.^[71–73]^ The formation of research teams is a natural requirement for the development of scientific research, as well as an effective way to address complex problems, promote academic innovation, and improve research efficiency and quality. In the future, the trend of research strength in this field should still strengthen the cooperation of multidisciplinary talents to better promote the development of disciplines, improve clinical effects, and promote international cooperation.

### 4.3. Research hotspot analysis and prospects

Based on the results of this study, the main research hotspots of non-pharmacological therapies for improving gait abnormalities after stroke can be divided into the following parts: the development of technology-assisted rehabilitation; the effectiveness of multimodal rehabilitation interventions; research on the mechanism of neuroplasticity. The rise of these research hotspots may be closely related to technological advances and changes in clinical requirements. The continuous development of technology has made more precise rehabilitation treatment possible, and the growing demand for personalized treatment in clinical practice has promoted the in-depth study of related research.

#### 4.3.1. The development of technology-assisted rehabilitation

With the rapid development of technology, technology-assisted rehabilitation has shown great potential in the treatment of poststroke gait abnormalities. Physical therapists have a wide range of rehabilitation equipment to choose from when deciding on rehabilitation plans in clinical practice, in addition to conventional methods such as manual physical therapy and occupational therapy. The application of technologies such as RAT, VR, FES, and TMS, has improved the accuracy, fun, and interactivity of rehabilitation treatment, greatly promoting the rehabilitation effect of patients.^[74]^

RAT has become an important area of research in poststroke gait rehabilitation.^[75]^ With the continuous advancement of robotic technology, RAT devices are increasingly used in gait rehabilitation.^[76]^ Various robots can assist with walking, including exoskeletons, end-effector robots, and lokomat.^[77]^ The principle of RAT is to assist patients with gait training through the external force from a mechanical device.^[78]^ Robotic technology not only provides precise and highly repeatable movement training but also meets the rehabilitation needs of different patients through personalized settings, and provides assistance and feedback when needed.^[79]^ Studies have shown that training walking ability through robots can improve standing balance, walking ability, stride length, and velocity.^[20,80,81]^ Royal Dutch Society for Physical Therapy guideline also suggests that RAT can help stroke patients who are unable to walk independently improve their velocity, walking distance, balance, and daily living abilities.^[82]^ The American Heart Association guideline also recommends robot-assisted exercise training combined with conventional treatment to improve motor function and mobility after stroke (class IIb, level A).^[83]^ A Cochrane review published in 2020 concluded that patients who are unable to walk independently during the first 3 months after a stroke event are more likely to benefit from RAT.^[84]^ Meanwhile, RAT is deemed safe as adverse events were not more frequent or severe in the intervention group. Although RAT shows great potential in gait rehabilitation, it also faces some challenges. First, the high cost of the equipment limits its widespread clinical application. Secondly, although existing research shows that RAT has a significant effect on gait improvement, its training frequency, long-term efficacy, and impact on patients’ quality of life still need to be validated through more clinical trials.

In addition, VR has also shown great potential in improving patient engagement and treatment outcomes.^[85]^ It uses computer technology to provide an immersive visual environment, allowing patients to practice specific tasks.^[86]^ Compared with real-world practice, VR training can provide more feedback on participants’ performance.^[22]^ Feedback is divided into internal feedback and external feedback. Internal feedback mainly includes somatosensory information, which is often impaired in stroke patients.^[87]^ External feedback refers to the feedback information provided by the external environment. VR can simulate real walking scenes during training. After training, therapists and patients can receive information on the execution results of the training task, helping patients improve gait control.^[88]^ Multiple systematic reviews have shown that VR can effectively improve lower limb balance, gait, and daily function in stroke patients.^[89–91]^ According to the American Heart Association guidelines, VR may be beneficial for the improvement of gain (class IIb, level B).^[83]^ Despite this, VR also has the problem of high equipment costs, and the long-term efficacy of VR training has not yet been verified by large-scale RCTs.^[88]^ How to reduce the cost of using VR, increase its popularity, and determine long-term efficacy in the future is a direction worth exploring.

FES is a rehabilitation technology that helps control foot drop and improve gait performance.^[92]^ Stroke patients typically experience reduced or absent dorsiflexion during initial contact and mid-swing.^[93]^ FES delivers an electrical current through transcutaneous electrodes to stimulate peripheral nerves, activate ankle dorsiflexion, and improve walking performance.^[94]^ Studies have found that FES increases the active ankle dorsiflexion angle during walking, improves walking ability, and enhances quality of life.^[95–97]^ However, the results of current systematic reviews have not yet provided evidence that FES improves gait stability and balance.^[98,99]^ In addition, due to skin resistance and the inconvenience of external devices, scholars have developed implantable functional electrical stimulation,^[100]^ but the results of a systematic review show that current research results cannot demonstrate the therapeutic benefits of implantable functional electrical stimulation for improving gait abnormality.^[101]^ In addition, other electrical stimulation technologies such as tDCS^[102–104]^ and neuromuscular electrical stimulation^[105–107]^ also play an important role in the rehabilitation of gait in stroke patients. Although the current systematic review results are skeptical about the clinical efficacy of FES, FES is widely used in clinical practice. In the future, by continuously optimizing electrical stimulation technology and parameter settings and matching the most beneficial stroke patient subgroups, it is expected to improve the application effect of FES and other electrical stimulation technologies in poststroke gait rehabilitation and provide evidence for further promotion and application of FES. In addition, with the continuous advancement of community rehabilitation and family rehabilitation concepts,^[108,109]^ the future development of portable FES devices will greatly facilitate patients’ gait rehabilitation.^[110]^

Another technology that has received much attention is TMS. TMS generates magnetic fields in specific areas of the cerebral cortex to regulate neuronal activity, thereby promoting neuroplasticity and improving gait function.^[111]^ Current research on TMS in the field of gait rehabilitation mainly focuses on the selection of stimulation areas and stimulation schemes.^[112]^ The selection of stimulation areas includes the dorsolateral prefrontal cortex, primary motor cortex, supplementary motor area, and cerebellum.^[113,114]^ The most commonly used low-frequency and high-frequency frequencies are 1 Hz and 10 Hz, and the stimulation course can be 2 to 3 weeks.^[115]^ Although some studies are skeptical about the effect of TMS on improving walking, balance, motor function, and daily living activities after stroke,^[50,116]^ the results of a systematic review have shown that TMS can effectively promote muscle strength, walking ability, and overall lower limb function recovery in stroke patients.^[117,118]^ Different stimulation frequencies, intensities, durations, and targets have different regulatory effects on neurons. Therefore, how to adjust these parameters according to the individual needs of patients will continue to be a research focus in the future. By continuously optimizing TMS technology and parameter settings, future research is expected to further enhance the application effect of TMS in poststroke gait rehabilitation.

#### 4.3.2. The effectiveness of multimodal rehabilitation interventions

In the past decade, multimodal rehabilitation interventions have shown great potential and efficacy in poststroke gait abnormality rehabilitation. Beyond traditional single therapies, they combine various equipment or methods like physical/occupational therapy,^[75,119–122]^ forming comprehensive plans. Included studies show combined treatments outperform monotherapy. Multimodal intervention emphasizes comprehensive advantages, especially for complex gait issues,^[123–125]^ and its effectiveness is a core hotspot. It aims for synergies between methods to maximize functional recovery.

A typical application is bodyweight support (BWS) with treadmill training.^[126]^ BWS eases lower limb burden, reduces fall risk, enabling safer exercises.^[127,128]^ Combined with treadmill training, it provides a stable environment and allows precise adjustment of intensity/parameters, accelerating gait improvement.^[129,130]^ A 2017 review found no significant difference in independent walking ability with/without BWS versus no treadmill training,^[131]^ but a recent review noted it improves early poststroke stride length, velocity,^[130]^ offering intensity hard to get traditionally.^[132]^ BWS combined with taichi,^[133]^ visual feedback,^[134]^ FES,^[135,136]^ or TMS^[137]^ also works. However, treadmill use is limited outside rehabilitation settings; therapists should assist transition to home/community self-rehabilitation.^[138]^

Besides BWS–treadmill, RAT–VR combination is key. RAT offers personalized training by controlling gait trajectory/force, providing support^[139,140]^; VR creates immersive environments with real-time feedback, boosting engagement.^[141,142]^ Their combination may optimize motor learning, promote neuroplasticity, improve function, and enhance participation.^[143,144]^ Zhang et al^[74]^ suggested it may best improve lower limb balance, but few combined studies exist. Another review found promising evidence for poststroke lower limb function but needs long-term follow-up.^[145]^ Future focus includes short/long-term efficacy, program selection, and effects across stroke stages.

FES combined with other technologies is important. For example, with treadmill training or RAT, it restores muscle strength/joint range and improves gait parameters,^[136,146,147]^ potentially becoming more comprehensive.^[21]^ Combining tDCS, spinal stimulation, neuromuscular electrical stimulation, RAT, mirror therapy, VR, or other stimulations also enhances walking ability.^[146,148–153]^

Multimodal methods address specific gait issues and overall function/mental health.^[154–156]^ Synergies enhance effects, improving symmetry, velocity, balance, reducing falls, and boosting daily activities, confidence, and quality of life. This highlights comprehensive intervention; clinicians should consider overall patient conditions.

It can be seen that interdisciplinary and multimodal rehabilitation strategies are gradually receiving attention, and gait rehabilitation is no longer dependent on a single treatment method. However, how to optimize the selection of interdisciplinary and multi technology rehabilitation programs and explore individualized rehabilitation programs for stroke patients are still unresolved issues. Therefore, personalized patient selection, rational allocation of multiple technical programs, and research on mid to long term rehabilitation efficacy in multimodal rehabilitation will still be a research hotspot in the future. In addition, in this study, we found that traditional Chinese medicine methods, such as taichi^[133]^ and acupuncture,^[157,158]^ were combined with rehabilitation technologies to improve hemiplegic gait and achieved certain results. In the future, we should also further explore the efficacy and mechanism of traditional medicine in gait rehabilitation.

#### 4.3.3. Research on the mechanism of neuroplasticity

Neuroplasticity refers to the ability of the central nervous system to partially or completely restore its original function by reorganizing and reconnecting neural networks after injury.^[159]^ Studies have found that the brain structure and functional connectivity of stroke patients change.^[160]^ After a stroke event, a spontaneous and natural neural reorganization process occurs, but individual recovery is incomplete and unpredictable.^[161,162]^ Therefore, understanding and promoting neuroplasticity is the key to improving rehabilitation outcomes, especially in the process of gait rehabilitation, where neuroplasticity plays a crucial role. Studies have shown that tDCS and foot drop stimulator treatment improve motor dysfunction in stroke patients, increase brain-derived neurotrophic factor, interleukin-10, and reduce interleukin-6 and tumor necrosis factor-alpha levels,^[163]^ demonstrating the effect of the combination of tDCS and foot drop stimulator on neuroplasticity biomarkers. Elsner study found^[164]^ that the improvement of balance ability in stroke patients by FES may be related to the regulation of oxidative stress markers. However, these results are from serological samples, and some patients may find it difficult to accept invasive procedures. In recent years, with the development of neuroimaging technology, a large number of noninvasive technologies, including functional magnetic resonance imaging, electroencephalography, and functional near-infrared spectroscopy, have emerged to facilitate the detection of neuroplasticity in rehabilitation technology.^[165]^ In order to accurately regulate and predict prognosis, the study of the brain mechanism of rehabilitation technology based on neuroplasticity and neuroimaging technology has received increasing attention in the field of poststroke gait rehabilitation and has gradually become one of the research hotspots.

Studies have shown that RAT can change the connection mode and strength of neural networks, increase activation of the lesion-side hemisphere, and improve gait performance.^[62]^ This change in neuroplasticity is not only reflected at the level of local neurons and synapses, but may also involve the functional reorganization of multiple brain networks.^[166–169]^ In addition, some noninvasive brain stimulation technologies can utilize neuroplasticity, including FES and TMS.^[113,170,171]^ These neurostimulation technologies increase the excitability of the primary motor cortex, premotor cortex, supplementary motor area, and sensorimotor cortex. In addition, a systematic review suggests that the combination of RAT and noninvasive brain stimulation may have a positive effect on walking rehabilitation, which may be related to neuroplasticity.^[172]^

The current neuroplasticity mechanism cannot reach a consistent conclusion in the field of poststroke gait rehabilitation. The main reasons include: most of the current studies focus on efficacy evaluation with less exploration of neural mechanisms, and heterogeneity of research results due to different stimulation schemes and equipment types of rehabilitation techniques. Based on the results of this study, it can be inferred that the research on the mechanism of neuroplasticity will continue to promote the development of the field of poststroke gait rehabilitation. With the advancement of neuroscience, how to maximize the rehabilitation effect by utilizing neuroplasticity should be explored in the future. This will not only help improve the rehabilitation effect, but also provide new ideas and methods for the formulation of personalized rehabilitation programs.

### 4.4. Limitations of research in this field

Based on the results of this study, the current research in this field still needs to be improved in the following aspects: large-sample, multicenter, double-blind, RCTs are still rare in the included literature, and standardized and rigorous RCTs should be designed to provide higher-level evidence for non-pharmacological therapies; although a core research force has been formed, the cooperation mode and research direction are relatively homogeneous, and multi-disciplinary, multi-field, and multi-institutional cooperation should be strengthened; efforts should be made to reduce the cost of rehabilitation equipment, improve its operability, and achieve popularization in more medical institutions; mechanism research is insufficient and current studies mostly focus on short-term treatment effects. There is a lack of research on how rehabilitation training promotes neuroplasticity; gait assessment tools are limited. Only a small number of researchers use 3-dimensional gait analysis systems, and most studies still lack the sensitivity and specificity of gait assessment.

## 5. Conclusion

Based on CiteSpace and VOSviewer, this study analyzed relevant research in this field since 2014, systematically reviewed the development trend of this field, and discussed and analyzed the research strength, literature sources, and research hotspots in this field. This study found the following research hotspots: the development of technology-assisted rehabilitation; the effectiveness of multimodal rehabilitation interventions; research on the mechanism of neuroplasticity. These focal points provide clear directions and guidance for future research, allowing scholars to choose more forward-looking and influential topics based on this. Secondly, through the analysis of high-volume journals, high-productivity authors, and highly cited literature, this study can help scholars quickly obtain important references and resources in this field.

This study also has certain limitations. Using only the WOS database as the source for literature analysis may not cover all relevant research. Although this study focuses on research trends within the past decade, new technologies and treatments in the field of rehabilitation medicine are developing rapidly. Some of the latest research and technology may not have been widely studied or fully published. This may prevent us from capturing the latest impact of these cutting-edge technologies on gait rehabilitation.

In conclusion, non-pharmacological therapies have significant potential in improving gait abnormalities after stroke. By improving the reliability and effectiveness of research, combined with personalized treatment options and emerging technologies, it is expected to further improve the overall level of gait rehabilitation and motor function in the future.

## Acknowledgments

Thanks to all who have worked hard on the writing of this article, as well as Director Yanguo Wang.

## Author contributions

**Conceptualization:** Xianggang Meng, Yuetong Li, Junfeng Zhang.

**Methodology:** Hao Chen, Yan Guo, Mengying Rong, Junfeng Zhang.

**Project administration:** Guiping Li, Yuzheng Du, Chen Li.

**Supervision:** Guiping Li, Yuzheng Du, Chen Li.

**Writing – original draft:** Hao Chen.

**Writing – review & editing:** Xianggang Meng, Junfeng Zhang.
